# A case of possible anaphylaxis to ASA and structurally unrelated NSAIDs

**DOI:** 10.1186/s13223-023-00830-2

**Published:** 2023-09-08

**Authors:** Sarah Edgerley, Harold Kim

**Affiliations:** 1 Division of Clinical Immunology and Allergy, Department of Medicine, Western University, London, ON Canada; 2 Department of Medicine, McMaster University, Hamilton, ON Canada

**Keywords:** Allergy, Anaphylaxis, Acetylsalicylic acid, NSAIDs

## Abstract

**Background:**

Non-steroidal anti-inflammatory drugs (NSAIDs) are one of the most commonly used classes of medications, and are among the leading causes of drug hypersensitivity. NSAIDs hypersensitivity reactions are classified by symptom involvement and NSAIDs subclass cross-reactivity. Reactions varying from cutaneous involvement to respiratory symptoms can be triggered by multiple NSAIDs subclasses. Anaphylaxis, while rare, can be induced by a single NSAID, with tolerability of other structurally unrelated subclasses. Reactions that fall outside of these traditional categories are deemed “blended reactions”. We report a unique case of possible anaphylaxis to acetylsalicylic acid (ASA) and ibuprofen, two structurally dissimilar NSAIDs, indicating a severe blended reaction outside of the typical NSAIDs hypersensitivity reaction categories.

**Case presentation:**

An otherwise healthy 45 year old woman was referred to the Allergy and Immunology clinic after developing acute onset dyspnea, lip swelling, and generalized urticaria with ibuprofen use requiring treatment with intramuscular epinephrine in the emergency department. She previously tolerated ibuprofen, naproxen, and acetaminophen and had no history of urticaria, angioedema, asthma, or nasal polyps. She underwent an oral challenge to ASA whereby she developed urticaria and throat irritation with rebound symptoms requiring 2 doses of intramuscular epinephrine. On subsequent visits she passed treatment dose acetaminophen and celecoxib challenges. She was counseled to avoid all other NSAIDs and ASA desensitization was offered should this medication be clinically indicated in the future.

**Conclusions:**

NSAIDs hypersensitivity reactions can be triggered by individual NSAIDs with tolerance of other subclasses or by multiple structurally unrelated NSAIDs due to COX-1 inhibition. Determining the type of reaction (NERD, NECD, NIUA, SNIUAA, or SNIDHR) allows for appropriate oral challenges and safe alternative therapy recommendations. However, not all clinical reactions fit perfectly into these categories. Patients may also develop blended reactions. Our case highlights a severe blended reaction to multiple unrelated NSAIDs, including likely anaphylaxis to ASA. We note the utility of drug provocation tests (DPTs) to identify safe alternative medication options, as well as the importance of performing DPTs in settings properly equipped to assess and manage severe hypersensitivity reactions including anaphylaxis.

## Background

Non-steroidal anti-inflammatory drugs (NSAIDs) are one of the most commonly used classes of medications worldwide and are among the leading causes of drug hypersensitivity reactions. [1, 2] NSAIDs hypersensitivity reactions are classified by symptom involvement and NSAIDs subclass cross-reactivity. Reactions varying from cutaneous involvement to respiratory symptoms can be triggered by multiple NSAIDs subclasses. [2] Anaphylaxis, while rare, can be induced by a single NSAID, with tolerability of other structurally unrelated subclasses. [2, 3] Reactions that fall outside of these traditional categories are deemed “blended reactions”. We report a unique case of possible anaphylaxis to acetylsalicylic acid (ASA) and ibuprofen, two structurally dissimilar NSAIDs, indicating a severe blended reaction outside of the typical NSAIDs hypersensitivity reaction categories.

## Case Presentation

An otherwise healthy 45 year old woman was referred to the Allergy and Immunology clinic after developing acute onset dyspnea, lip swelling, and generalized urticaria with ibuprofen use requiring treatment with intramuscular epinephrine in the emergency department. She previously tolerated ibuprofen, naproxen, and acetaminophen without reaction or adverse event and had no history of urticaria, angioedema, asthma, or nasal polyps. There were no other identifiable cofactors including alcohol consumption, acute illness, or exercise.

Her symptoms were reproducible on graded drug provocation test (DPT) to ibuprofen. Shortly after her final dose (cumulative 200 mg), she developed diffuse urticaria. High dose H1-antihistamines was given, however her symptoms quickly progressed to subjective throat tightness and irritation. Her vital signs and physical examination remained unchanged. Epinephrine 0.5 mg was administered intramuscularly and symptoms quickly resolved.

**Fig. 1:**
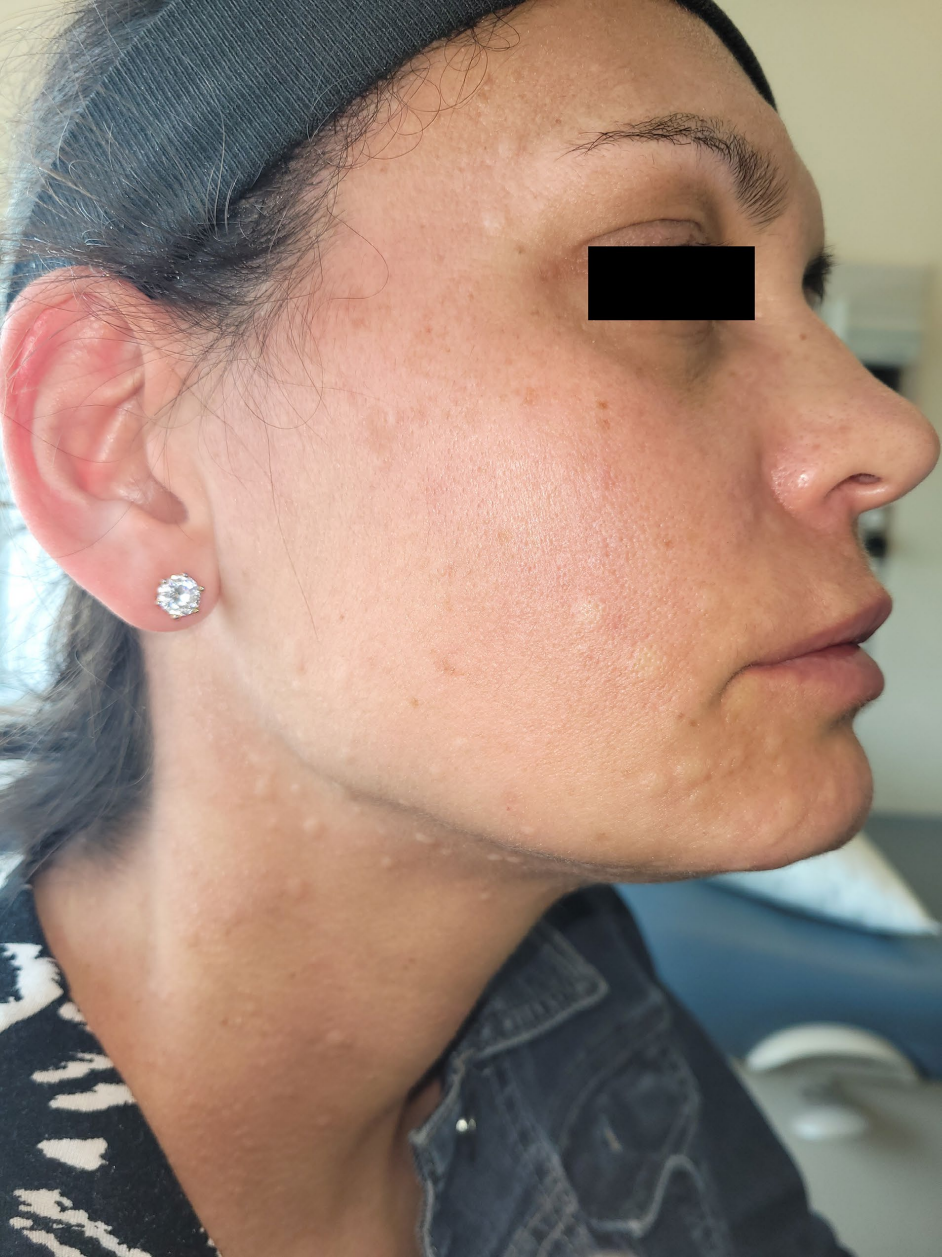
Acute onset urticaria of the patient’s face and neck

**Fig. 2:**
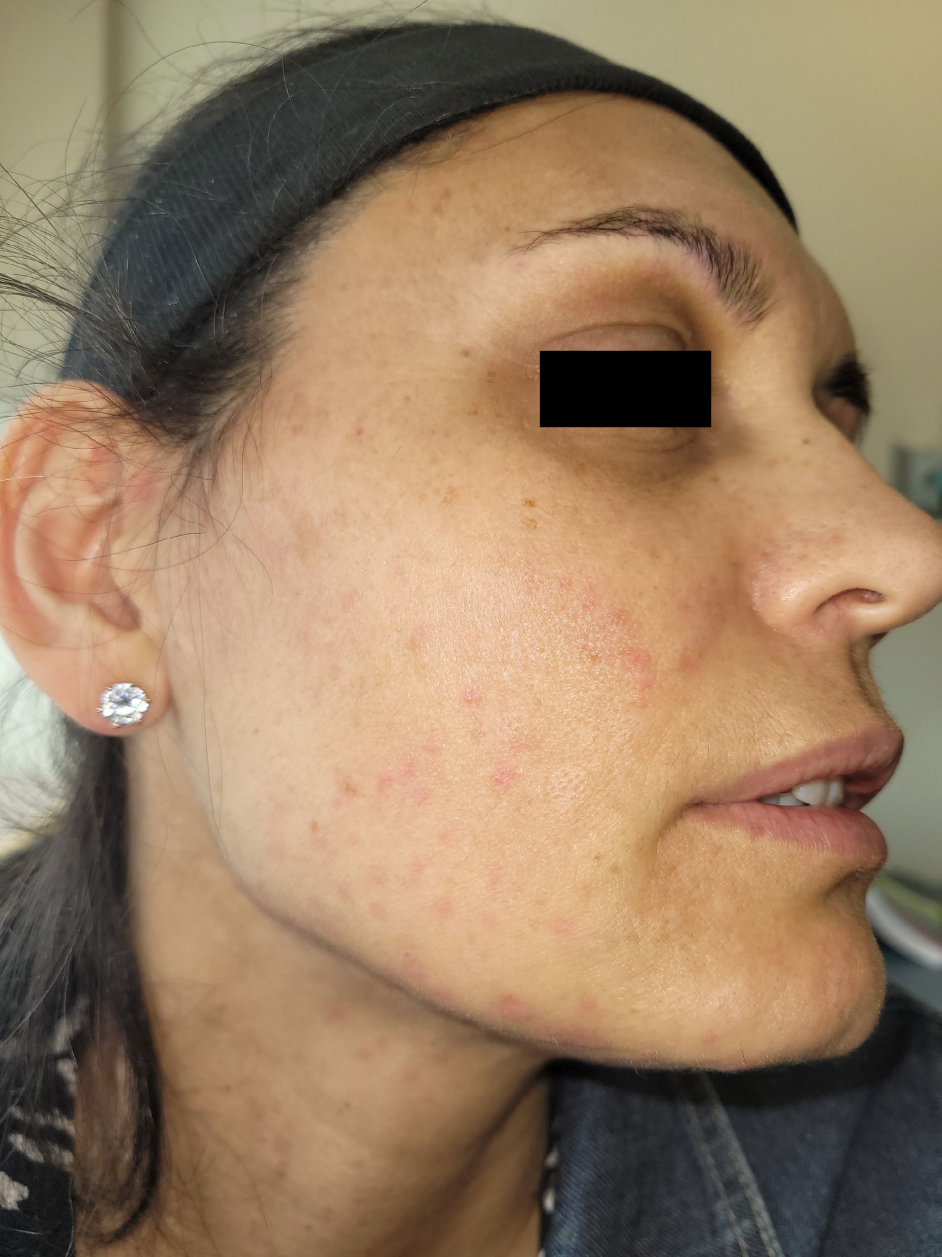
Demonstrable clinical improvement in the patient’s urticaria 5 minutes after epinephrine administration

**Fig. 3:**
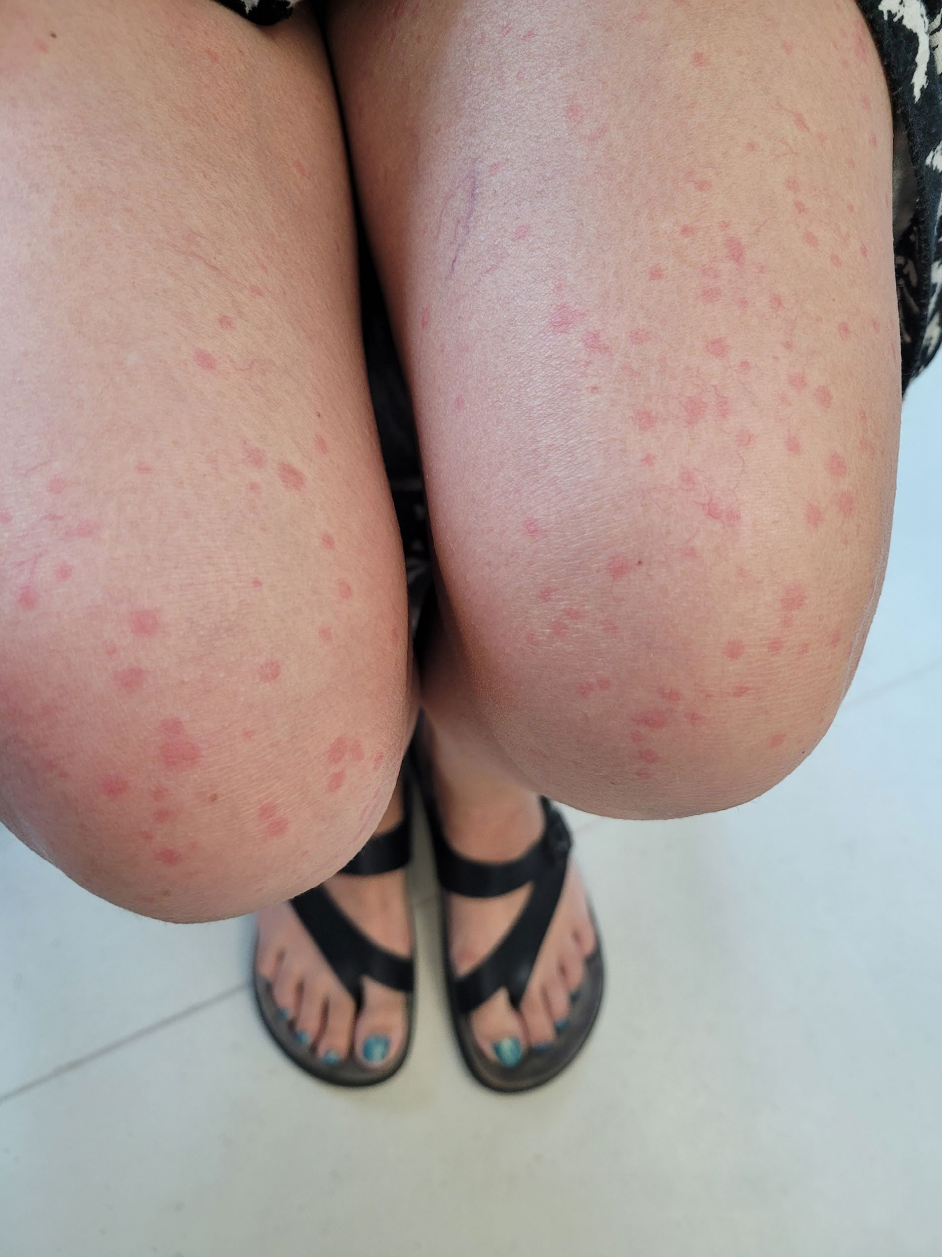
New onset generalized flat, erythematous, blanchable, non-pruritic rash as seen on the patient?s thighs

She returned to clinic for a graded DPT to ASA. Doses were given at 30 min intervals. She tolerated her first 2 steps (5 mg, 60 mg) without incident. 15 min after her third dose (100 mg), the patient reported subjective pruritus of her neck without objective overlying skin changes. 10 min after this assessment she developed prominent urticaria of the face and neck (Fig. 1), audible voice hoarseness/change in voice quality, and subjective throat swelling and irritation. Repeat vital signs were unchanged from pre-challenge measurements. Epinephrine 0.5 mg was administered as well as prednisone 50 mg and high dose H1-antihistamines. Her symptoms improved within 5 min (Fig. 2), and completely resolved after 1 h of close observation. 15 min after symptom resolution (almost 2 h after consuming her last dose of ASA), the patient developed a new rash, clinically distinct from her previously noted urticaria. The rash was erythematous, blanchable, non-pruritic, and flat (Fig. 3). She had no associated cardiac, respiratory, or gastrointestinal involvement. In context of her progressive symptoms, despite previous medical therapy, she was administered additional epinephrine 0.3 mg. Her skin lesions improved shortly afterwards and did not recur.

At her subsequent appointments she successfully passed both graded DPTs to acetaminophen (cumulative 650 mg) as well as celecoxib (cumulative 200 mg). She was advised to avoid all other NSAIDs, to wear a medical alert bracelet, and to carry epinephrine auto injectors. ASA desensitization was advised if indicated in the future for cardioprotective or cerebrovascular indications.

## Discussion and conclusions

The NSAIDs hypersensitivity reactions are categorized into 5 groups based on symptoms, mechanisms, and cross-reactivity: NSAIDs-exacerbated respiratory disease (NERD), NSAIDs-exacerbated cutaneous disease (NECD), NSAIDs-induced urticaria/angioedema (NIUA), single NSAID-induced urticaria/angioedema and anaphylaxis (SNIUAA), and single NSAID-induced delayed hypersensitivity reactions (SNIDHR). [2, 4].

The first three phenotypes are cross intolerance reactions whereby symptoms can be triggered by multiple structurally unrelated NSAIDs across more than one subclass. [1] (Table 1). These reactions are mediated by cyclooxygenase 1 (COX-1) inhibition blocking prostaglandin synthesis from arachidonic acid and promoting leukotriene production. [4, 5].

**Table 1:** Classification of NSAIDs by chemical structure [1, 7, 11]

Chemical Group	Examples
Salicylic Acid Derivatives	ASA Sodium salicylate Salsalate Sulfasalazine
Para-aminophenol	Acetaminophen
Propionic Acid Derivatives	Ibuprofen Naproxen Ketoprofen Flurbiprofen Fenoprofen
Acetic Acid Derivatives	Diclofenac Ketorolac Indomethacin Sulindac
Enolic Acid Derivatives	Oxicams: Piroxicam Meloxicam Tenoxicam Pyrazolones: Phenylbutazone
Fenamic Acid Derivatives	Mefenamic Acid Meclofenamic Acid
Selective COX-2 Inhibitors	Celecoxib Etoricoxib Parecoxib
Alkalones	Nabumetone
Sulphonanilide Derivatives	Numesulide

1. NERD manifests as respiratory involvement (dyspnea, cough, wheeze, nasal congestion, rhinorrhea) in patients with underlying airways disease including asthma, nasal polyps, and rhinosinusitis. [2].
2. NECD manifests as wheals/urticaria and/or angioedema in patients with a history of chronic spontaneous urticaria (CSU). [2] Up to 30% of CSU patients experience exacerbation of their underlying cutaneous disease with NSAIDs use. [6, 7].
3. NIUA manifests as wheals/urticaria and/or angioedema in otherwise healthy patients without an underlying history of CSU. [2, 4].

The final two groups are selective reactor reactions whereby clinical symptoms are caused by a single NSAID or single NSAIDs subclass, with tolerability of structurally different subclasses. [1] These reactions are immune mediated and not related to degree of COX-1 inhibition. [5].

4. SNIUAA is characterized by an immediate hypersensitivity reaction, likely mediated by a specific IgE antibody. [1, 4] Patients may develop urticaria, angioedema, or anaphylaxis. [2].
5. SNIDHR typically occurs 24–48 h after drug administration and involves cutaneous symptoms such as exanthems, fixed drug eruptions, or severe cutaneous adverse reactions. [2] Reactions are presumed mediated by specific T-cell responses. [1].

However, not all clinical reactions fit perfectly into the above traditional categories. Patients can also experience blended reactions, which are estimated to represent up to 28% of NSAIDs hypersensitivity reactions. [5] Symptoms range from isolated gastrointestinal involvement, to multisystemic reactions, to anaphylaxis. [5] These blended reactions have been further divided into 4 subclasses: [5].

I: development of cutaneous symptoms and rhinitis/asthma.

II: development of cutaneous symptoms and glottis edema.

III: development of cutaneous symptoms, rhinitis/asthma, and glottis edema.

IV: development of a combination of gastrointestinal symptoms with cutaneous symptoms, and/or rhinitis/asthma.

Patients who develop multisystemic involvement, including glottis edema and throat tightness, can be clinically indistinguishable from anaphylaxis. [5].

When assessing patients with possible NSAIDs hypersensitivity it is important to clarify and classify the type of reaction as this will have significant clinical and treatment implications. Available skin testing and laboratory testing have limited diagnostic or predictive value. [1, 4] DPTs remain the gold standard for evaluation and diagnosis, and can determine cross-reactivity and safe alternative medications. [1, 4] Acute reactions during challenges are managed similarly to other hypersensitivity reactions including discontinuation of the inciting medication, administration of epinephrine in cases of anaphylaxis, and consideration of supportive therapies including H1-antihistamines or steroids. [4] NERD reactions are typically treated with bronchodilators or leukotriene receptor antagonists. [4].

In the US, ibuprofen and naproxen are the most common NSAIDs associated with SNIUAA. [4] DPT to ASA may be considered as a first step as successful oral challenge to ASA confirms tolerance of structurally unrelated NSAIDs without requiring repeat exposure to the inciting medication. [4] Patients with SNIDHR should also continue to avoid the culprit agent but will tolerate other NSAIDs. [4] For cross-reactive reactions including NERD, NECD, and NIUA, patients should avoid both the triggering as well as cross-reacting COX-1 inhibitors. [2] NSAIDs avoidance remains the mainstay of treatment. [4] Several DPTs may be required to establish the diagnosis and to determine cross-reactivity and safe medication alternatives in order to expand patients’ available therapeutic options. [4].

Alternative therapies for treatment of fever, inflammation, and pain should be identified. [1] Weak COX-1 inhibitors, acetaminophen, and COX-2 inhibitors can be considered. (Table 2). However, weak COX-1 inhibitors may induce symptoms in up to 25% of patients with NIUA, especially when higher doses are used. [2, 8, 9] Cyclooxygenase 2 (COX-2) inhibitors such as celecoxib are typically well tolerated. [4, 10] Formal DPTs to these alternative medications can be considered prior to prescription particularly in patients with a history of severe reaction. [2, 4] In the case of our patient she passed DPTs to treatment doses of acetaminophen and celecoxib.

**Table 2:** Classification of NSAIDs by degree of COX inhibition [11, 13]

COX Inhibition	Examples	COX-1 Inhibition	COX-2 Inhibition
Strong COX-1 Inhibition	ASA, Ibuprofen, Naproxen (non-selective NSAIDs)	++	At high doses
Weak COX-1 Inhibition	Acetaminophen	Partial (at high doses)	Preferential (at low doses)
Preferential COX-2 Inhibition	Meloxicam	Partial (at high doses)	+
Selective COX-2 Inhibition	Celecoxib	-	++

The prevalence of ASA hypersensitivity has been estimated to be 0.5–1.9%.^11^ Although rare, anaphylactic type reactions to ASA have also been reported. [3] This adds additional clinical complications in treating patients with cardiovascular or cerebrovascular disease where no suitable alternative agent can be used. [3, 12] Fortunately, ASA desensitization can be performed. [12].

## Conclusions

NSAIDs are one of the most frequently used classes of medications and most common triggers of drug hypersensitivity reactions. Symptoms can be induced by individual NSAIDs with tolerance of other subclasses, or by multiple structurally unrelated NSAIDs due to COX-1 inhibition. Determining the type of reaction (NERD, NECD, NIUA, SNIUAA, or SNIDHR) allows for appropriate DPTs and safe alternative therapy recommendations. However, a large proportion of patients experience blended reactions, with symptoms that fall outside of these traditional categories. Our case highlights one example of a severe blended reaction to multiple unrelated NSAIDs, ASA and ibuprofen. We additionally note the utility of DPTs to identify safe alternative medication options, as well as the importance of performing DPTs in settings properly equipped to assess and manage severe hypersensitivity reactions including anaphylaxis. [3].

## Data Availability

Not applicable.

## List of abbreviations

ASA: Acetylsalicylic acid
COX-1: Cyclooxygenase 1
COX-2: Cyclooxygenase 2
CSU: Chronic spontaneous urticaria
DPT: Drug provocation test
NECD: NSAIDs-exacerbated cutaneous disease
NERD: NSAIDs-exacerbated respiratory disease
NIUA: NSAIDs-induced urticaria/angioedema
NSAIDs: Non-steroidal anti-inflammatory drugs
SNIDHR: Single NSAID-induced delayed hypersensitivity reaction
SNIUAA: Single NSAID-induced urticaria/angioedema or anaphylaxis
